# The macrophage polarization–ferroptosis axis as a therapeutically targetable immunometabolic framework in periodontitis

**DOI:** 10.3389/fimmu.2026.1904152

**Published:** 2026-08-11

**Authors:** Zhengrong Li, Xin Liu, Jianing Song, Xiangtao Ma, Yuning Liu, Taohong Liu, Mingxuan Wu

**Affiliations:** 1 Department of Periodontology (II), School and Hospital of Stomatology, Hebei Key Laboratory of Stomatology, Hebei Technology Innovation Center of Oral Health, Hebei Medical University, Shijiazhuang, Hebei, China; 2 Department of Laser Medicine, School and Hospital of Stomatology, Hebei Key Laboratory of Stomatology, Hebei Technology Innovation Center of Oral Health, Hebei Medical University, Shijiazhuang, Hebei, China

**Keywords:** ferroptosis, host modulation, immunometabolism, iron homeostasis, macrophage polarization, oxidative stress, periodontitis

## Abstract

Periodontitis is a chronic inflammatory disease sustained by microbial dysbiosis, maladaptive host immunity, oxidative stress, and progressive destruction of periodontal supporting tissues. Macrophage polarization and ferroptosis have usually been discussed as separate pathological processes, but increasing evidence suggests that they may be functionally connected within the periodontal inflammatory microenvironment. This review evaluates the macrophage polarization–ferroptosis axis as an emerging and therapeutically targetable immunometabolic framework in periodontitis. We summarize how pro-inflammatory cytokine networks, iron retention, reactive oxygen species accumulation, lipid metabolic remodeling, antioxidant defense failure, ferroptosis-associated danger signaling, defective efferocytosis, and extracellular vesicle-mediated communication may contribute to the proposed crosstalk between macrophages and ferroptosis-vulnerable periodontal cells. This framework helps explain how periodontal lesions may progress from microbial-triggered inflammation to self-sustaining tissue destruction and impaired repair. Potential therapeutic opportunities include relatively higher-priority host-modulatory nodes, such as macrophage reprogramming and ferroptosis-relevant redox regulation, as well as candidate or emerging approaches, including iron modulation, biomaterial-assisted local delivery, exosome-based approaches, and adjunctive photobiomodulation. However, most current evidence remains preclinical, evidence strength varies across mechanistic nodes, cell-type specificity is incompletely resolved, and clinically validated intervention strategies are lacking. Future translation will require spatially resolved human validation, standardized macrophage and ferroptosis phenotyping, mechanism-matched target-engagement biomarkers, and locally deliverable host-modulatory platforms. Refining this framework may support mechanism-based host-modulatory strategies for periodontitis.

## 1 Introduction

Periodontitis is a chronic inflammatory disease initiated by plaque biofilm-associated microbial dysbiosis and sustained by maladaptive host responses. Rather than arising solely from bacterial burden, periodontal tissue destruction reflects a complex interplay among persistent inflammatory activation, oxidative stress, aberrant immune regulation, and impaired reparative capacity (1–3). Within this pathological network, the progression from reversible inflammation to irreversible attachment loss and alveolar bone resorption is strongly shaped by the local immune microenvironment, in which macrophages are key regulators. Through dynamic functional reprogramming, macrophages coordinate pathogen sensing, inflammatory amplification, osteoimmune regulation, tissue clearance, and repair-associated remodeling (4–6). Although the classical M1/M2 paradigm does not fully capture the heterogeneity of macrophage states *in vivo*, it remains a useful conceptual framework for understanding how maladaptive polarization contributes to persistent periodontal inflammation. In general, sustained M1-skewed responses exacerbate cytokine production, oxidative stress, and osteoclastogenic signaling, whereas reparative macrophage programs are more closely associated with inflammatory resolution and tissue regeneration (7–9).

Concurrently, ferroptosis has emerged as a mechanistically important form of regulated cell death in inflammatory and degenerative disorders. Defined by iron-dependent lipid peroxidation and the collapse of antioxidant defenses, ferroptosis provides a conceptual framework linking redox imbalance, iron dysregulation, and tissue injury (10). This mechanism is particularly relevant to periodontitis, where reactive oxygen species accumulation, inflammatory bleeding, heme release, disrupted iron handling, and impaired antioxidant buffering collectively create a microenvironment conducive to ferroptotic damage (11, 12).

Macrophage polarization and ferroptosis should therefore be considered as potentially interconnected, rather than entirely independent, pathological processes in periodontitis. Available evidence indicates that macrophage inflammatory activation, oxidative stress, iron-related dysregulation, and ferroptosis-associated molecular alterations can coexist within the periodontal inflammatory microenvironment (11, 13). In this context, macrophages may contribute to local cytokine production, iron-handling changes, and redox imbalance that favor ferroptotic injury, whereas ferroptotic or insufficiently cleared cells may generate secondary inflammatory cues that sustain macrophage activation and interfere with reparative reprogramming (13, 14). However, direct evidence demonstrating causal and bidirectional interactions between macrophage polarization and ferroptosis in periodontal tissues remains limited. Therefore, in this review, we use the macrophage polarization–ferroptosis axis as a conceptual and hypothesis-generating immunometabolic framework, rather than as a fully validated causal pathway.

**Figure 1:**
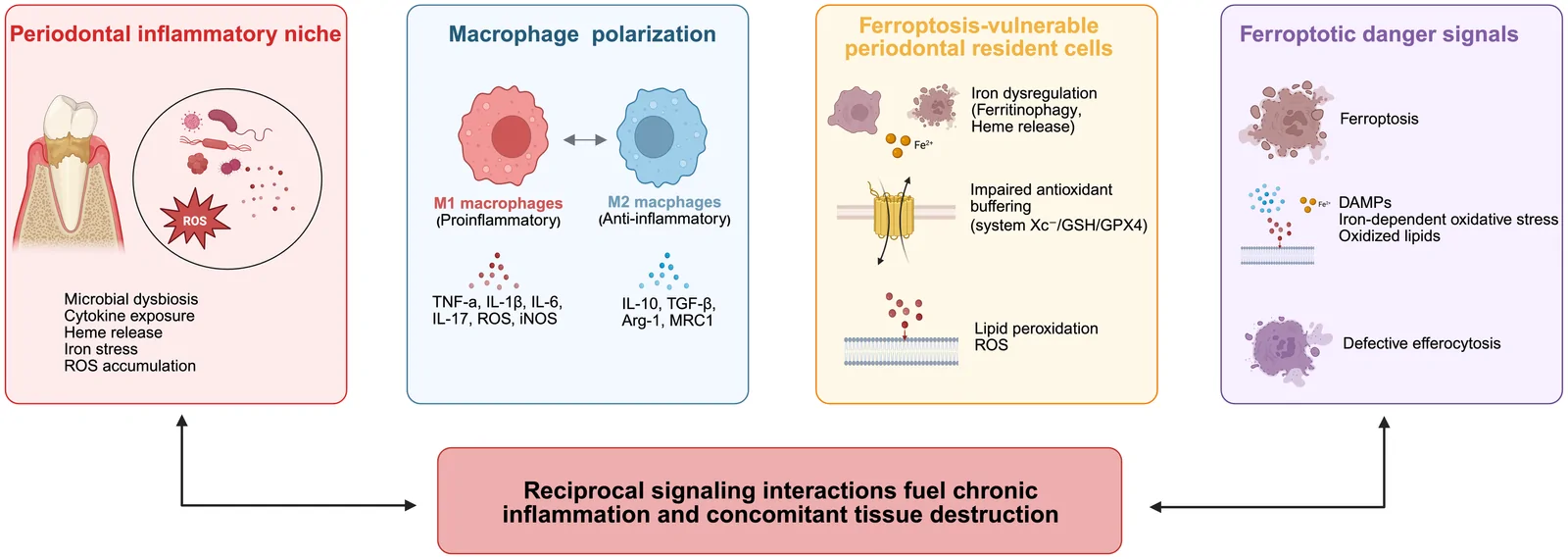
The macrophage polarization–ferroptosis axis in periodontitis. The periodontal inflammatory niche, shaped by microbial dysbiosis, cytokine exposure, heme release, iron dysregulation, and ROS accumulation, promotes maladaptive macrophage reprogramming and ferroptotic vulnerability in periodontal resident cells. M1 macrophages produce pro-inflammatory cytokines and ROS, whereas M2 macrophages contribute to anti-inflammatory, repair-associated, and resolution-related responses. These immune–redox signals may sensitize periodontal ligament cells, epithelial cells, and osteoblast-lineage cells to ferroptotic injury through iron dysregulation, lipid peroxidation, GSH depletion, and impairment of the system Xc^−^/GSH/GPX4 axis. Ferroptotic danger signals, iron-dependent oxidative stress, oxidized lipids, and defective efferocytosis may reinforce macrophage activation, potentially sustaining inflammatory feedback and periodontal tissue destruction. DAMPs, damage-associated molecular patterns; GPX4, glutathione peroxidase 4; GSH, glutathione; IL, interleukin; M1, pro-inflammatory macrophage phenotype; M2, anti-inflammatory macrophage phenotype; ROS, reactive oxygen species; system Xc^−^, cystine/glutamate antiporter; TNF-α, tumor necrosis factor-α; TGF-β, transforming growth factor-β; Arg-1, arginase 1; iNOS, inducible nitric oxide synthase; MRC1, mannose receptor C-type 1.

We discuss how maladaptive macrophage reprogramming intersects with ferroptotic susceptibility through cytokine-driven iron remodeling, redox–lipid failure, damage-associated molecular pattern signaling, efferocytosis, emerging extracellular vesicle-mediated communication, and cell-type-specific effects. We further evaluate therapeutically targetable and emerging translational opportunities involving macrophage reprogramming, ferroptosis-relevant redox and lipid peroxidation modulation, iron–redox control, nanomaterials, exosome-based approaches, local delivery systems, and adjunctive photobiomodulation. By organizing current evidence according to evidence source, causal strength, bidirectional plausibility, and translational limitations, this review aims to bridge periodontal immunopathology with broader concepts in immunometabolism, regulated cell death, and host-directed therapy. The conceptual framework of the proposed macrophage polarization–ferroptosis axis in periodontitis is summarized in **Figure 1**.

## 2 The periodontal inflammatory microenvironment as a ferroptosis-permissive and macrophage-reprogramming niche

Periodontal lesions provide a combined microbial, inflammatory, oxidative, and iron-enriched microenvironment that may simultaneously reshape macrophage function and increase ferroptotic susceptibility in resident periodontal cells.

### 2.1 Macrophage heterogeneity and maladaptive polarization in periodontitis

Macrophages in periodontal tissues are neither static nor homogeneous. Instead, they comprise both tissue-resident and recruited subsets whose phenotypes are dynamically shaped by microbial stimulation, inflammatory cytokines, metabolic stress, and tissue damage-associated signals (11, 15). Although the M1/M2 dichotomy oversimplifies the full *in vivo* spectrum of macrophage states, it remains a useful conceptual framework for understanding how maladaptive functional reprogramming contributes to the progression of periodontal disease (16, 17).

In periodontitis, macrophage responses are often skewed toward a persistent pro-inflammatory state, characterized by increased production of IL-6, TNF-α, IL-1β, and other mediators that promote leukocyte recruitment, osteoclastogenic signaling, and oxidative stress. In contrast, reparative macrophage functions associated with inflammatory resolution, matrix remodeling, and regenerative support are often insufficiently sustained (18, 19). The pathological significance of this imbalance lies not only in the amplification of inflammation itself but also in its ability to reshape the local metabolic and redox milieu, thereby increasing tissue susceptibility to secondary injury pathways (20).

From a therapeutic perspective, macrophage heterogeneity suggests that indiscriminate anti-inflammatory suppression may be less effective than context-dependent reprogramming strategies designed to restore immune balance while preserving reparative potential (16, 21).

### 2.2 Why periodontal lesions are permissive to ferroptosis

Periodontal lesions create a microenvironment that is highly permissive to ferroptotic injury. Persistent pathogen-derived stimulation sustains ROS generation and inflammatory activation, while recurrent bleeding, heme release, and disrupted local iron homeostasis promote the accumulation of redox-active iron. Concurrently, chronic inflammation drives lipid remodeling and compromises antioxidant defenses, thereby lowering the threshold for iron-dependent lipid peroxidation (11, 22, 23).

In this context, ferroptosis is relevant not merely as a recognized form of regulated cell death, but also because its core determinants—oxidative overload, iron dysregulation, membrane lipid susceptibility, and impaired antioxidant defense—are all plausible features of the periodontal inflammatory niche (24). Consequently, periodontal ligament cells, epithelial cells, osteoblast-lineage cells, and immune cells may exhibit differential susceptibility to ferroptotic damage depending on cell type, inflammatory intensity, and disease stage (25, 26).

The significance of ferroptosis in periodontitis should therefore be understood within its disease-specific context: it may serve as a mechanistic bridge linking an oxidative and iron-enriched lesion microenvironment to tissue dysfunction, persistent inflammation, and impaired repair.

### 2.3 Linking immune dysregulation to ferroptotic injury

The link between immune dysregulation and ferroptotic injury in periodontitis is biologically compelling. Macrophages regulate cytokine signaling, ROS production, iron redistribution, and the clearance of dying cells, thereby shaping the susceptibility of surrounding tissues to ferroptosis (17, 27). Conversely, ferroptotic cells may release danger-associated signals, oxidized lipids, and lysophosphatidylcholine-related cues that further amplify inflammatory activation and destabilize the periodontal microenvironment (22).

This proposed relationship suggests that macrophage polarization and ferroptosis should be viewed not as parallel pathological events, but as interconnected components of a broader immunometabolic network (28–30). This perspective provides a more integrated framework for understanding why inflammatory tissue destruction persists in certain periodontal lesions despite partial control of the microbial trigger.

## 3 Proposed mechanistic interfaces between macrophage polarization and ferroptosis in periodontitis

**Figure 2:**
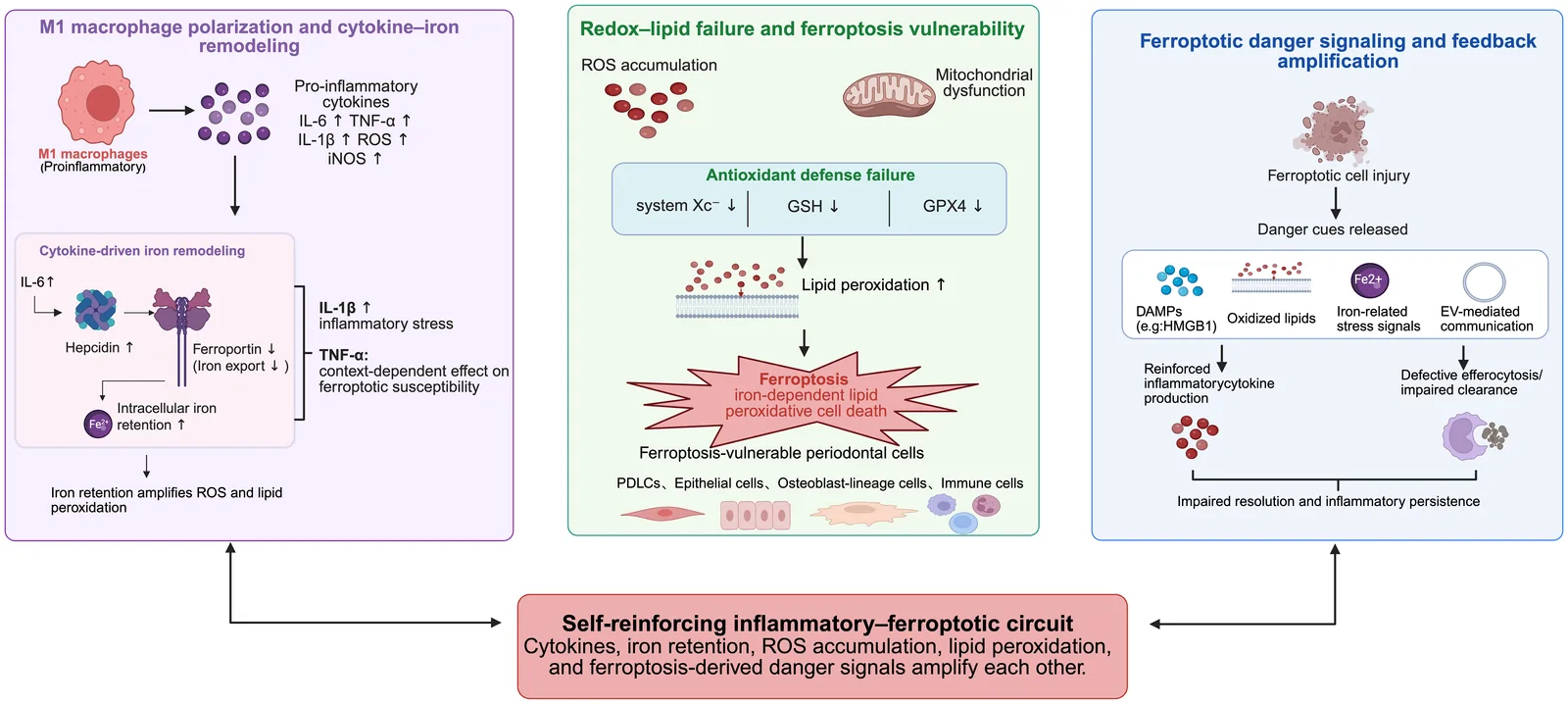
Mechanistic overview of proposed macrophage polarization–ferroptosis interactions in periodontitis. The schematic summarizes potential cytokine-driven iron remodeling, ROS accumulation, lipid peroxidation, antioxidant defense failure, ferroptotic danger signaling, defective efferocytosis, and extracellular vesicle-mediated communication within the periodontal inflammatory microenvironment. These interactions should be interpreted as a conceptual framework rather than as definitively proven causal pathways. The evidence source and causal strength for each proposed interface are summarized in **Table 1A**. DAMPs, damage-associated molecular patterns; EVs, extracellular vesicles; GPX4, glutathione peroxidase 4; GSH, glutathione; HMGB1, high mobility group box 1; IL, interleukin; iNOS, inducible nitric oxide synthase; M1, pro-inflammatory macrophage phenotype; PDLCs, periodontal ligament cells; ROS, reactive oxygen species; system Xc^−^, cystine/glutamate antiporter; TNF-α, tumor necrosis factor-α.

In periodontitis, dysregulated macrophage polarization and ferroptotic injury may be interconnected through several inflammatory, metabolic, redox, and clearance-related interfaces, rather than discrete pathological modules. Persistent microbial challenge, oxidative stress, inflammatory bleeding, and iron dysregulation create a lesional microenvironment in which macrophages shape inflammatory tone while also modulating the metabolic and redox conditions that govern ferroptotic susceptibility. Conversely, ferroptotic injury may intensify macrophage activation through danger signaling, defective clearance, and altered intercellular communication (12, 28, 29). However, the strength of evidence differs substantially among these proposed mechanisms. At present, periodontal evidence more strongly supports the coexistence of macrophage inflammatory activation, oxidative stress, iron-related dysregulation, and ferroptosis-associated molecular alterations than it supports direct bidirectional causality between macrophage polarization and ferroptosis. Therefore, in this section, “crosstalk” is used as a conceptual term to describe a proposed interaction network that integrates direct periodontal observations with mechanistic insights extrapolated from non-periodontal inflammatory, metabolic, and degenerative disease models. The following sections organize this proposed network into five mechanistic interfaces: cytokine-driven iron remodeling, ROS–lipid peroxidation–antioxidant failure, ferroptotic danger signaling and defective efferocytosis, extracellular vesicle-mediated communication, and cell-type- or stage-dependent effects. **Figure 2** provides a conceptual synthesis of these proposed interactions. To improve transparency regarding the evidence base, **Table 1A** summarizes the proposed directionality, evidence source, causal strength, current confidence, and key limitations for each macrophage polarization–ferroptosis interface discussed in this section. The key unresolved questions that should be addressed before clinical translation are summarized in **Box 1**.

**Table 1A:** Directionality, evidence source, evidence level, and key limitations of proposed macrophage polarization–ferroptosis interfaces in periodontitis.

Proposed interface	Direction	Evidence source	Evidence level	Key limitation
Cytokine-driven iron remodeling, including IL-6–hepcidin–ferroportin signaling	Macrophage → ferroptotic vulnerability	Periodontal and periodontal-adjacent studies support selected components, including inflammatory cytokine activity, hepcidin regulation, iron-related alterations, and ferroptosis-associated periodontal injury. Several links remain extrapolated from non-periodontal iron-inflammation models. Representative references (11, 22, 33, 34, 36, 42, 45, 46).	Mostly correlative/inferential; moderate confidence	A complete periodontal causal chain linking macrophage-derived cytokines to hepcidin/ferroportin remodeling, iron retention, ferroptosis, and tissue destruction remains unproven.
ROS–lipid peroxidation–antioxidant failure	Macrophage/redox inflammation → ferroptotic vulnerability	Periodontitis-specific studies support ROS accumulation, antioxidant impairment, lipid peroxidation, GPX4/SLC7A11- or NRF2-related changes, and ferroptosis-associated periodontal injury. Representative references (11, 25, 26, 42, 45–49, 54).	Correlative plus preclinical functional evidence; moderate confidence	Direct evidence that macrophage-derived ROS is sufficient and necessary to induce ferroptosis in defined periodontal resident cells remains limited.
Ferroptotic DAMPs, HMGB1-related signaling, and defective efferocytosis	Ferroptosis → macrophage activation/inflammatory persistence	Ferroptotic DAMP release and HMGB1-related macrophage activation are mainly supported by non-periodontal ferroptosis models. Periodontal studies support DAMP signaling, macrophage clearance, and impaired resolution more broadly. Representative references (29, 55–59, 61, 62, 65, 66).	Mostly inferential in periodontitis; low-to-moderate confidence	Direct periodontal evidence showing that ferroptotic periodontal cells sustain macrophage activation through RAGE/AGER- or TLR4-related signaling remains limited.
EV-mediated remote regulation	Potentially bidirectional	Periodontal evidence supports EV-mediated regulation of macrophage behavior and inflammatory responses. EV mechanisms involving ferroptosis-related pathways are mainly extrapolated from non-periodontal models. Representative references (67–79).	Emerging/hypothesis-generating; low confidence	Disease-relevant EV cargo, donor-cell identity, recipient-cell specificity, and ferroptosis-related endpoints remain insufficiently defined in periodontitis.
Cell-type-specific and stage-dependent effects	Context-dependent	Periodontal and periodontal-adjacent evidence supports cell-dependent ferroptosis-associated markers and macrophage responses. Stage-dependent effects are mostly inferred from destructive, resolving, and regenerative contexts. Representative references (25, 26, 42, 45, 80–90).	Moderate for PDLCs/fibroblasts and epithelial barriers; moderate/context-dependent for osteoblast-lineage cells; limited and predominantly periodontal-adjacent for osteoclast-related compartments; mostly inferred for macrophage ferroptotic death and stage-dependent causality	Spatially resolved human validation is needed to link macrophage states, ferroptosis-vulnerable cells, and lesion stage.

Box 1 **Key unresolved questions before clinical translation.**

1. Which periodontal cell populations are truly vulnerable to ferroptosis *in vivo*?
2. Are macrophages upstream drivers, downstream responders, or amplifiers of ferroptotic injury?
3. Which macrophage programs—not simply M1/M2 labels—causally regulate iron retention, lipid peroxidation, defective efferocytosis, and inflammatory persistence?
4. Which biomarkers can identify lesion phenotypes or patient subgroups most likely to benefit from ferroptosis-aware host-modulatory therapy?
5. Can local delivery platforms maintain mechanistically relevant local concentrations without impairing antimicrobial defense, wound healing, or regenerative remodeling?
6. How should intervention timing differ across destructive, resolving, and regenerative phases?
7. Which molecular endpoints should be paired with clinical measures such as probing depth, clinical attachment level, bleeding on probing, alveolar bone preservation, and regenerative gain?

### 3.1 Cytokine-driven iron remodeling and ferroptosis susceptibility

One major route through which polarized macrophages may influence ferroptotic vulnerability in periodontitis is the cytokine-driven remodeling of local iron handling and membrane susceptibility to oxidation. Pro-inflammatory mediators released by M1-skewed macrophages, particularly IL-6, TNF-α, and IL-1β, may not only amplify inflammation but also shift the lesional microenvironment toward intracellular iron retention, lipid metabolic dysregulation, and increased sensitivity to oxidative membrane injury. Among these pathways, IL-6–hepcidin–ferroportin signaling is a particularly relevant candidate pathway because it provides a plausible mechanistic link between inflammatory activation and iron sequestration, thereby potentially lowering the threshold for ferroptosis in adjacent periodontal cells (13, 31–35).

Within the inflammatory periodontal niche, sustained IL-6 signaling may contribute to local hepcidin upregulation and reduced ferroportin-dependent iron export, favoring intracellular iron retention (36, 37). Such iron retention can promote oxidative stress and lipid peroxidation (38, 39). In parallel, IL-1β can amplify inflammatory stress and promote lipid oxidation, whereas the effect of TNF-α on ferroptotic susceptibility appears to be context dependent (40, 41). Collectively, cytokines derived from polarized macrophages may shape ferroptosis-related vulnerability in both resident periodontal cells and infiltrating immune cells (42–44).

From a disease perspective, cytokine-associated iron remodeling may provide a mechanistic bridge linking persistent inflammation to periodontal tissue destruction and bone loss (22, 42). Accordingly, M1-skewed macrophage activity may be viewed not only as a reflection of ongoing inflammation, but also as a potential contributor to ferroptosis-related vulnerability within periodontal lesions (37, 45). Whether this form of inflammatory iron remodeling operates uniformly across periodontal ligament cells, epithelial cells, osteoblast-lineage cells, and immune subsets remains unclear and requires cell-type-specific functional testing (22, 46). Thus, cytokine-driven iron remodeling may represent a key interface through which inflammatory macrophage states increase ferroptotic vulnerability in periodontal lesions.

#### 3.1.1 Evidence status

Evidence source: Periodontal and periodontal-adjacent evidence supports several components of this pathway, including ferroptosis-related signatures in human gingival crevicular fluid during periodontitis progression, IL-6-induced hepcidin responses in periodontitis-related inflammatory conditions, P. gingivalis-associated macrophage polarization involving hepcidin regulation in chronic apical periodontitis, and ferroptosis-related injury in periodontal fibroblasts or osteogenic lineages (11, 22, 34, 36, 42, 45, 46). However, several mechanistic links used to construct the full IL-6–hepcidin–ferroportin–ferroptosis pathway are supported mainly by non-periodontal or disease-general models, including studies of macrophage–ferroptosis interactions, iron-immune crosstalk, hepcidin-driven inflammation, lysosomal iron-triggered ferroptosis, and cytokine-dependent ferroptotic susceptibility in other inflammatory diseases (31, 32, 35, 37, 38, 41, 43, 44). Causal strength: Taken together, these studies support biological plausibility and partial periodontal relevance, but they do not yet establish a complete causal chain in periodontal tissues whereby M1-skewed macrophage-derived cytokines activate IL-6–hepcidin–ferroportin signaling, cause iron retention in defined periodontal cell populations, and induce ferroptosis leading to tissue destruction. Key gap: Future studies should directly test macrophage-selective cytokine perturbation, hepcidin–ferroportin expression, labile iron accumulation, lipid peroxidation, and ferroptosis rescue in co-culture systems, animal models, and spatially resolved human periodontal lesions.

### 3.2 ROS, lipid metabolism, and redox failure as a shared hub

Reactive oxygen species, lipid metabolic remodeling, and antioxidant failure may constitute a shared redox-sensitive interface linking macrophage polarization to ferroptotic vulnerability (38, 46). Macrophage activation reshapes the redox landscape of periodontal lesions through sustained ROS generation and inflammatory signaling, whereas ferroptosis represents the terminal outcome of unchecked iron-dependent lipid peroxidation in cells that can no longer maintain redox homeostasis (45). In this sense, macrophage polarization and ferroptosis may converge within the same redox-sensitive microenvironment, thereby potentially contributing to a self-reinforcing cycle of oxidative stress, membrane damage, and tissue dysfunction (43, 44).

During periodontitis, persistent periodontal pathogen-associated stimulation can sustain ROS generation and progressively disrupt local redox homeostasis (12, 47). Once antioxidant buffering capacity is exceeded, oxidative stress can promote membrane lipid peroxidation, deplete GSH-dependent defenses, and impair mitochondrial function, thereby increasing susceptibility to ferroptosis (12, 48). This vulnerability may be further amplified when the system Xc^−^/GSH/GPX4 axis is suppressed or when nuclear factor erythroid 2-related factor 2 (NRF2)-centered antioxidant programs fail to adequately restrain lipid peroxide accumulation (25, 26). Altered phospholipid remodeling, particularly through acyl-CoA synthetase long-chain family-mediated lipid remodeling, may further sensitize periodontal resident cells to ferroptotic injury under chronic inflammatory and oxidative conditions (49, 50).

ROS signaling and macrophage polarization appear closely associated within a shared inflammatory and redox network in periodontitis. On the one hand, excessive ROS can reinforce pro-inflammatory macrophage programming through redox-sensitive signaling and metabolic reprogramming (51, 52). On the other hand, M1-skewed macrophages are associated with sustained cytokine output and a more oxidative tissue milieu in periodontitis (52, 53). This shared redox–inflammatory hub may help explain why anti-inflammatory, antioxidant, and ferroptosis-relevant interventions can show convergent protective effects in experimental periodontitis. These findings support the rationale for exploring combined strategies that target inflammation, oxidative stress, and ferroptotic vulnerability (48, 53, 54).

#### 3.2.1 Evidence status

Evidence source: Periodontitis-specific studies support the presence of ROS accumulation, antioxidant impairment, lipid peroxidation, mitochondrial stress, and ferroptosis-associated molecular changes in periodontal cells or experimental periodontal lesions. For example, periodontitis-relevant stimulation has been reported to induce ROS accumulation, GSH depletion, GPX4 reduction, lipid peroxidation, and ACSL4-related lipid susceptibility in periodontal ligament fibroblasts, while experimental periodontitis models and periodontal tissue studies have implicated the NRF2/GPX4, SLC7A11/GPX4, and related antioxidant-defense pathways in ferroptosis-associated periodontal injury (11, 25, 26, 42, 45–49, 54). Evidence linking macrophage activation, ROS amplification, and ferroptosis is biologically plausible and is supported by periodontal-adjacent or non-periodontal inflammatory models, including studies of macrophage ferroptosis and ROS-dependent inflammatory injury (43–45, 51, 52). Causal strength: Compared with the IL-6–hepcidin–ferroportin pathway, the redox–lipid peroxidation–antioxidant failure interface has stronger periodontitis-specific support. However, direct periodontal evidence remains limited for the complete causal sequence in which M1-skewed macrophages generate sufficient ROS or redox pressure to induce ferroptosis in defined periodontal resident cells. Therefore, this mechanism should be interpreted as a well-supported redox convergence point, but not yet as a fully proven macrophage-driven ferroptosis pathway in periodontal tissues. Key gap: Future studies should use macrophage-conditioned media, macrophage-specific redox manipulation, co-culture systems, lipid peroxidation readouts, mitochondrial stress assays, and rescue experiments with ferroptosis inhibitors or restoration of the system Xc^−^/GSH/GPX4 and NRF2/GPX4 axes to determine whether macrophage-derived oxidative stress is sufficient and necessary to drive cell-type-specific ferroptosis in periodontitis.

### 3.3 Ferroptotic DAMPs, defective efferocytosis, and inflammatory persistence

In periodontitis, ferroptotic cell death is unlikely to be entirely immunologically silent, although its inflammatory consequences in periodontal tissues remain incompletely defined. Ferroptotic cells may release HMGB1 and other lipid peroxidation-associated danger cues, thereby potentially converting cell death into renewed inflammatory stimulation (29, 55). HMGB1-related danger signaling may engage receptors such as the receptor for advanced glycation end products (RAGE/AGER) and, depending on cellular context, toll-like receptor 4 (TLR4), thereby amplifying secondary inflammatory signaling and cytokine production rather than resolution (29, 56, 57). In periodontal tissues already exposed to persistent microbial challenge, this process, together with insufficient efferocytic clearance, could hinder timely resolution and favor inflammatory persistence, although direct periodontal validation of ferroptosis-derived DAMP signaling remains limited (58, 59).

When efferocytic clearance is insufficient, the tissue-level pathological impact of ferroptosis may be amplified (60, 61). Under homeostatic conditions, macrophages clear dying cells and tissue debris, thereby limiting secondary inflammatory signaling and promoting tissue repair and resolution (60, 62). In a periodontal inflammatory microenvironment, particularly under persistent microbial challenge and oxidative stress, macrophage efferocytic capacity may be attenuated (63–65). Ferroptotic or incompletely cleared dying-cell remnants may then persist within lesions, prolong danger signaling, and favor inflammatory persistence (61, 66). From this perspective, ferroptosis may be viewed not only as a cell-autonomous death pathway, but also as a process with potential consequences for tissue-level inflammatory persistence when local clearance fails (63, 64, 66).

#### 3.3.1 Evidence status

Evidence source: Evidence that ferroptotic cells can release HMGB1 and other danger-associated signals is supported mainly by mechanistic studies outside periodontitis (29, 55, 66). HMGB1-related inflammatory amplification through receptors such as RAGE/AGER and, depending on cellular context, TLR4 is supported by oral wound-healing, periodontal, and non-periodontal studies, but direct periodontal ferroptosis-specific causality remains untested (29, 56, 57, 66). Periodontal studies support the broader relevance of DAMP-related signaling, macrophage-mediated clearance, and impaired inflammatory resolution without establishing ferroptosis-derived DAMPs as drivers of macrophage activation in periodontal lesions (57, 58, 61, 62). Causal strength: The current evidence for this interface is therefore mainly inferential in periodontitis. Ferroptotic DAMP release, HMGB1-dependent macrophage activation, and efferocytosis failure have experimental support in broader inflammatory or regulated-cell-death contexts (29, 55, 56, 59, 60, 63–66) and in selected periodontal studies of HMGB1/RAGE signaling or macrophage-mediated clearance (57, 58, 61, 62), but a complete periodontal causal chain—ferroptotic periodontal cells releasing HMGB1 or oxidized lipid cues, activating macrophages through RAGE/AGER- or TLR4-related signaling, and thereby sustaining periodontal inflammation—has not yet been fully demonstrated. Key gap: Future studies should test whether ferroptosis blockade reduces HMGB1 release, RAGE/AGER or TLR4 signaling, and macrophage inflammatory activation in periodontal models. In parallel, studies should determine whether oxidized lipid signals directly alter macrophage polarization and whether restoring macrophage efferocytosis interrupts the proposed ferroptosis–DAMP–macrophage feed-forward loop in periodontal lesions.

### 3.4 Emerging extracellular vesicle-mediated communication: an extrapolated regulatory layer

Beyond soluble cytokines and direct cell–cell interactions, extracellular vesicles (EVs), particularly exosomes, may represent an emerging and incompletely validated regulatory layer linking macrophage polarization to ferroptotic responses (67, 68). Evidence from non-periodontal inflammatory, metabolic, and degenerative disease models suggests that macrophage-derived EVs can modulate iron–redox homeostasis, antioxidant defense, lipid peroxidation, and inflammatory signaling in recipient cells (68, 69). Although direct evidence in periodontitis remains limited, this mechanism is biologically plausible within the highly interactive periodontal microenvironment and should currently be regarded as an emerging hypothesis rather than an established periodontal mechanism (70).

The relevance of this pathway lies in the disease-specific composition and functional impact of EV cargo. Depending on their polarization state, macrophage-derived exosomes may deliver microRNAs (miRNAs), proteins, and metabolic regulators capable of modulating pathways related to SLC7A11, GPX4, mitochondrial metabolism, or inflammatory signal transduction. In principle, this would enable polarized macrophages to regulate ferroptotic susceptibility at a distance, thereby extending their influence beyond direct cytokine exposure (71–73). Conversely, EVs from gingival or periodontal stem-cell populations can modulate macrophage behavior or regenerative signaling and may thereby provide a potential feedback route into the proposed framework (74–78).

Specific candidate cargo should be interpreted cautiously. In non-periodontal models, macrophage- or MSC-derived EV cargo such as Apoc1, THBS1, ficolin B, or cargo that affects SLC7A11/glutathione synthesis has been linked to ferroptosis regulation (67–69, 73, 79). By contrast, periodontium-relevant EV studies more directly implicate engineered macrophage EVs, gingival or periodontal stem-cell EVs, and cargos such as miR-143-3p or miR-31-5p in macrophage polarization, inflammatory regulation, or regenerative signaling rather than ferroptosis-specific target engagement (70, 74–78).

#### 3.4.1 Evidence status

Evidence source: Direct periodontal evidence supports the broader relevance of EV-mediated regulation of macrophage behavior, periodontal inflammation, and regenerative responses through engineered macrophage exosomes and EVs derived from gingival or periodontal stem-cell populations (70, 74–78). However, direct evidence that macrophage-derived or periodontal cell-derived EVs regulate ferroptosis in periodontitis remains limited. Much of the evidence linking EV cargo to ferroptosis-related pathways, including SLC7A11, GPX4, mitochondrial metabolism, lipid peroxidation, and iron–redox homeostasis, is extrapolated from non-periodontal inflammatory, metabolic, degenerative, or oncological models (67–69, 71–73). Causal strength: The current evidence for EV-mediated macrophage–ferroptosis crosstalk in periodontitis is therefore low and mainly hypothesis-generating. Periodontal EV studies support EV-dependent modulation of macrophage polarization and inflammatory signaling, while non-periodontal studies support the ability of EV cargo to regulate ferroptosis-related pathways. However, a complete periodontal causal chain—polarization-specific macrophage EV cargo altering ferroptosis in defined periodontal recipient cells, or ferroptotic periodontal cell-derived EVs reciprocally reprogramming macrophages—has not yet been demonstrated. Key gap: Future studies should define disease-relevant EV cargo, donor-cell identity, recipient-cell specificity, and functional ferroptosis endpoints in periodontal models. Priority readouts should include SLC7A11, GPX4, ACSL4, lipid peroxidation, mitochondrial stress, labile iron, and rescue experiments using EV inhibition, EV cargo manipulation, or ferroptosis-relevant pathway modulation.

### 3.5 Cell-type-specific and stage-dependent effects

The biological significance of the macrophage polarization–ferroptosis axis is unlikely to be uniform across periodontal cell types and may also vary between destructive and reparative contexts (80–83). Ferroptosis in periodontal ligament cells, epithelial cells, or osteoblast-lineage cells is likely to exacerbate tissue dysfunction and impair repair, whereas ferroptosis modulation within osteoclast-related pathways or inflammatory macrophage subsets may produce more context-dependent effects (80–82, 84). Likewise, mechanisms that intensify injury in chronic destructive lesions may not operate in the same way during early inflammatory phases or subsequent reparative attempts (83).

These distinctions have important therapeutic implications. The value of ferroptosis modulation cannot be defined in absolute terms, as the consequences of intervention are likely to depend on cellular context, lesional microenvironment, and therapeutic timing. Therefore, ferroptosis inhibition should not be assumed to be universally beneficial across all periodontal cell populations or disease stages. Similar caution applies to macrophage targeting: a shift in macrophage phenotype may be beneficial in one lesional context, yet insufficient or differently effective in another (85, 86). Future studies should therefore combine cell-resolved functional analyses with lesion-stage classification to determine how specific macrophage subsets and ferroptosis-vulnerable cell populations contribute to tissue destruction or repair (87, 88).

A concise cell-type reading of the current evidence is that ferroptosis-related injury is relatively better supported in periodontal ligament fibroblasts/PDLCs and epithelial barrier cells, moderate or context-dependent in osteoblast-lineage cells, limited and predominantly periodontal-adjacent in osteoclast-related compartments, and still mostly inferred for macrophage ferroptotic death as a driver of periodontal outcomes. These distinctions are reflected in **Table 1B** and should guide selection of endpoint panels in future studies (25, 26, 42, 45, 81–83, 89, 90).

**Table 1B:** Cell-type-specific consequences of ferroptosis in periodontal tissues and current evidence levels.

Cell type	Predicted consequences of ferroptosis	Current evidence level	Representative references
PDLCs/periodontal fibroblasts	Reduced viability, impaired matrix homeostasis, and diminished osteogenic or regenerative capacity; likely to aggravate tissue breakdown and defective repair.	Moderate; periodontitis-specific cellular and preclinical evidence.	(26, 42, 80, 89)
Gingival/periodontal epithelial cells	Barrier-junction injury and amplification of inflammatory and redox stress.	Moderate; periodontitis-specific cellular evidence.	(25, 81)
Osteoblast-lineage cells	Impaired osteogenic differentiation, mineralization, or bone-regenerative capacity.	Moderate but context-dependent; periodontal and periodontal-adjacent evidence.	(22, 82, 83)
Osteoclast-related compartments	Potential alteration of osteoclastogenesis and bone-resorption dynamics; direction depends on cellular and lesion context.	Limited and predominantly periodontal-adjacent; direct periodontal causal evidence is lacking.	(84, 90)
Macrophages	Potential effects on inflammatory persistence, DAMP release, efferocytosis, and polarization; causal impact on periodontal outcomes remains uncertain.	Mostly inferential for macrophage ferroptotic death in periodontitis; periodontal-adjacent evidence is stronger.	(29, 43–45, 55, 58–66)

Evidence levels were assigned qualitatively from source type, periodontal relevance, and causal strength; they are intended as a transparent synthesis rather than a formal grading system. Direct representative references are listed in **Tables 1A**, **B**, with fuller context in Sections 3.1–3.5..

#### 3.5.1 Evidence status

**Evidence source:** Available periodontal and periodontal-adjacent evidence supports cell-dependent differences in ferroptosis-associated markers and macrophage responses, particularly across periodontal ligament cells, epithelial barriers, osteoblast-lineage cells, osteoclast-related compartments, and immune-cell populations (80–84). However, most studies evaluate individual cell types, selected ferroptosis markers, or isolated inflammatory conditions rather than spatially resolving the macrophage polarization–ferroptosis axis across intact human periodontal lesions. Evidence supporting stage-dependent effects is even more limited and is largely inferred from differences between destructive inflammation, resolution, and regenerative contexts (83, 85–88). Causal strength: Evidence for ferroptosis-associated injury appears more developed for periodontal resident structural cells than for macrophage subsets or osteoclast-related compartments. However, the functional consequences of ferroptosis modulation remain context dependent, and current data do not yet establish whether ferroptosis inhibition, macrophage reprogramming, or combined modulation would be beneficial across all periodontal disease stages. Key gap: Future studies should combine spatial transcriptomics, multiplex immunostaining, single-cell or cell-resolved phenotyping, ferroptosis-rescue assays, macrophage subset mapping, and lesion-stage classification. This approach will be necessary to determine which cell populations are ferroptosis-vulnerable, which macrophage states are functionally linked to ferroptotic injury, and when ferroptosis inhibition, macrophage reprogramming, or combined host modulation is most appropriate.

## 4 Therapeutically targetable nodes and emerging translational opportunities within the macrophage polarization–ferroptosis axis

**Figure 3:**
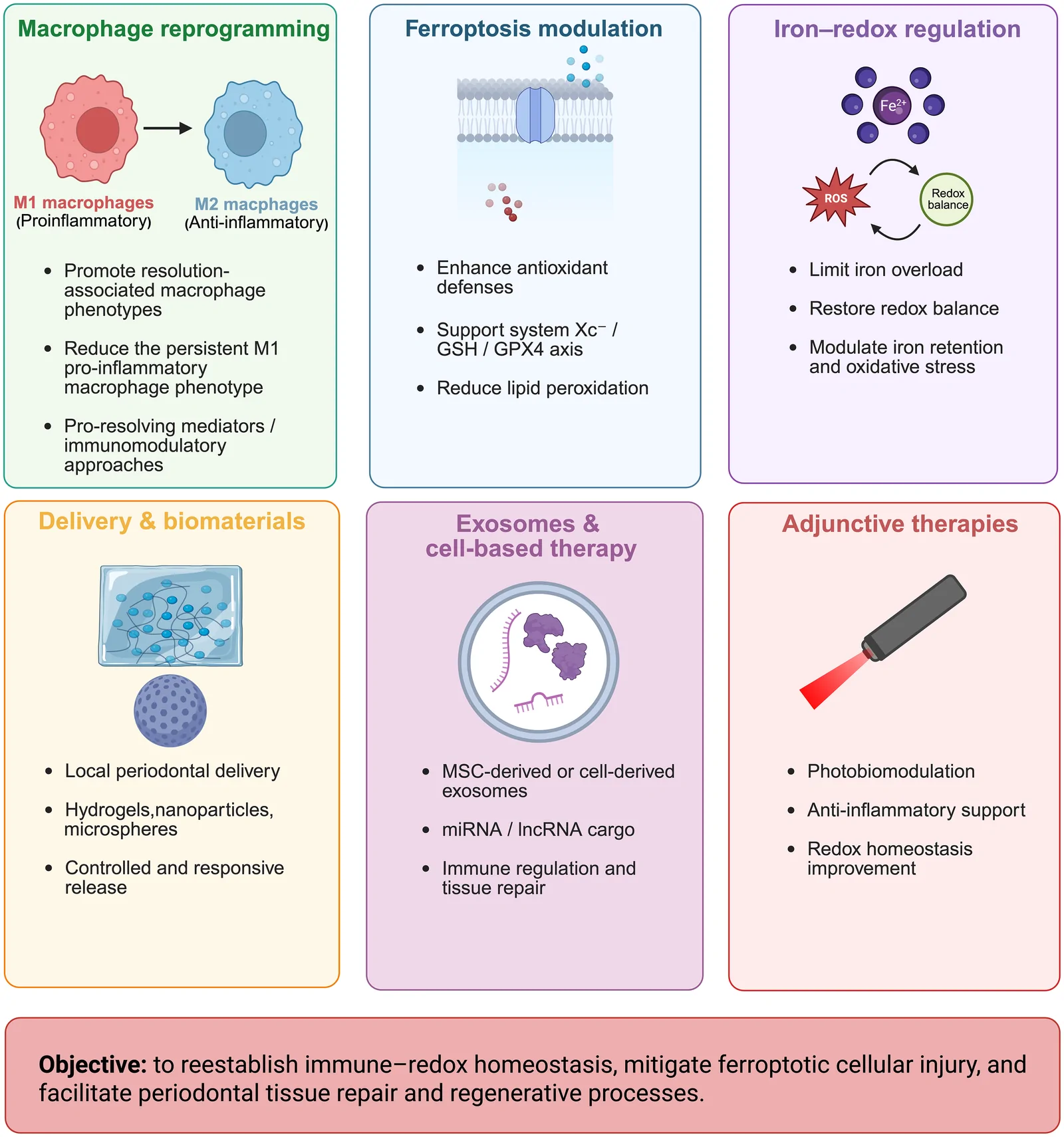
Therapeutically targetable and emerging translational strategies within the proposed macrophage polarization–ferroptosis framework in periodontitis. The schematic summarizes candidate approaches, including macrophage reprogramming, ferroptosis-relevant redox and lipid peroxidation modulation, iron modulation, biomaterial-assisted local delivery, exosome-based approaches, and adjunctive photobiomodulation. These approaches may help modulate immune–redox imbalance, reduce ferroptosis-related cellular injury, and support periodontal repair and regeneration, but remain largely preclinical and require further periodontal validation. GPX4, glutathione peroxidase 4; GSH, glutathione; lncRNA, long noncoding RNA; M1, pro-inflammatory macrophage phenotype; M2, anti-inflammatory macrophage phenotype; miRNA, microRNA; MSC, mesenchymal stem cell; ROS, reactive oxygen species; system Xc^−^, cystine/glutamate antiporter.

From a translational perspective, the macrophage polarization–ferroptosis axis may be considered therapeutically targetable because it encompasses multiple nodes linking immune imbalance, redox dysfunction, and tissue destruction. Rather than treating inflammation, oxidative stress, ferroptotic injury, and regenerative failure as separate pathological events, an axis-based framework suggests that coordinated modulation of the lesional microenvironment may provide adjunctive host-modulatory opportunities in periodontitis. This concept is especially relevant to periodontitis, in which chronic destructive lesions may be insufficiently controlled by single-node intervention alone (33, 91, 92). However, the therapeutic relevance of individual nodes should be interpreted according to evidence strength, target specificity, and feasibility of local periodontal delivery. Macrophage reprogramming and redox–lipid peroxidation control may represent relatively higher-priority intervention nodes, whereas iron modulation, efferocytosis restoration, biomaterial-assisted delivery, EV-based approaches, and adjunctive photobiomodulation should currently be viewed as candidate or emerging strategies requiring further periodontal validation. Target engagement may be assessed using mechanism-matched macrophage, ferroptosis-related redox/lipid peroxidation, iron-handling, inflammatory, and tissue-repair readouts. Based on the mechanistic framework described above, **Figure 3** summarizes the major therapeutically targetable and emerging translational strategies proposed within the macrophage polarization–ferroptosis axis in periodontitis.

Timing should also be stage matched: anti-inflammatory and redox-stabilizing approaches may be most relevant during active destructive inflammation, macrophage reprogramming and local delivery may be most rational after initial debridement when microbial burden has been reduced, and biomaterial-, exosome-, or photobiomodulation-supported strategies may be better positioned as adjuncts during resolution or regenerative phases. These assignments remain provisional and require direct clinical testing.

### 4.1 Reprogramming macrophage polarization

Reprogramming macrophage polarization may represent an upstream strategy to interrupt the pathological link between persistent inflammation and ferroptotic vulnerability in periodontal lesions (17, 93). Rather than simply suppressing inflammation, this approach aims to shift the periodontal microenvironment from a destructive, M1-skewed state toward a resolution-competent and repair-supportive profile (17, 19, 93). By attenuating the burden of pro-inflammatory cytokines, oxidative amplification, and maladaptive osteoimmune signaling, macrophage reprogramming may indirectly reduce ferroptotic susceptibility in surrounding periodontal cells while preserving a microenvironment conducive to tissue regeneration (17, 19, 47, 93).

A range of candidate approaches has been explored to achieve this effect, including natural bioactive compounds, biomaterial-based immunomodulators, and host-modulatory nanosystems (94). The common rationale underlying these strategies is not simply to reduce inflammatory mediator levels, but to remodel the lesional microenvironment at an upstream regulatory level (95). Within this framework, successful macrophage reprogramming may dampen IL-6-, TNF-α-, and IL-1β-dominated signaling, alleviate oxidative stress, and restore a local milieu more conducive to resolution and regeneration (96).

The therapeutic value of macrophage-targeted interventions therefore lies not only in correcting immune imbalance but also in mitigating the inflammatory and metabolic conditions that favor ferroptosis. This makes macrophage reprogramming an attractive entry point for adjunctive host-modulatory therapy, particularly when combined with local delivery systems capable of sustaining immune modulation within the periodontal pocket. In practice, such approaches may be most appropriate after microbial burden has been reduced by initial periodontal therapy, when the goal shifts from acute inflammatory control toward resolution and repair. Greater selectivity will be required to account for macrophage heterogeneity *in vivo* and ensure mechanistic precision for clinical translation.

### 4.2 Ferroptosis-relevant redox and lipid peroxidation modulation

Direct modulation of ferroptotic susceptibility may constitute a second therapeutic entry point within this axis (48). In periodontitis, such interventions may act by restoring antioxidant defenses, limiting iron-driven lipid peroxidation, stabilizing redox homeostasis, or modulating iron handling in susceptible cell populations (97). Candidate targetable nodes include system Xc^−^, GPX4, NRF2-dependent antioxidant programs, and related pathways that govern ROS accumulation and membrane lipid oxidation (26). Importantly, the therapeutic rationale is not to suppress cell death indiscriminately, but to selectively reduce destructive ferroptotic injury in contexts where it contributes to persistent inflammation and impaired tissue repair (30).

Several bioactive compounds with antioxidant and potential ferroptosis-modulating properties are relevant in this context. Agents such as quercetin, silybin, resveratrol, and curcumin may activate NRF2/heme oxygenase-1 (HO-1)-associated defense pathways, buffer oxidative overload, suppress inflammatory ROS amplification, and modulate lipid peroxidation-related injury (97–101). Because many of these agents exert broad antioxidant and anti-inflammatory effects, their periodontal relevance should be interpreted as ferroptosis-relevant rather than ferroptosis-specific unless supported by mechanism-matched readouts such as GPX4 restoration, SLC7A11 regulation, lipid peroxidation reduction, labile iron modulation, or ferroptosis-rescue assays. Within this framework, their therapeutic relevance may extend beyond generic antioxidant protection by helping to limit the redox and lipid peroxidation conditions that favor ferroptotic injury, thereby preserving the viability and function of structural periodontal cells (30).

At the same time, ferroptosis-relevant intervention should not be equated with indiscriminate ferroptosis inhibition (48). The biological consequences of such modulation are likely to vary according to target cell type and disease stage (102). Inhibition of ferroptosis may be protective in periodontal ligament cells, epithelial barrier cells, or osteoblast-lineage cells, whereas more selective or context-dependent modulation may be required in osteoclast-related pathways or inflammatory macrophage subsets (30, 81, 84). Accordingly, the future of ferroptosis-aware periodontal intervention lies in selective, lesion-aware modulation rather than nonspecific blockade (48, 84, 102). At present, much of this therapeutic promise remains mechanistically suggestive rather than clinically validated in human periodontitis.

### 4.3 Nanomaterials and local delivery systems

In periodontitis, the therapeutic potential of nanomaterials and local delivery systems may lie not only in enhancing drug retention within periodontal pockets but also in enabling more localized and controlled modulation of the macrophage polarization–ferroptosis axis (103). Compared with systemic administration, local platforms may increase tissue exposure, minimize off-target effects, and facilitate combinatorial delivery of immunomodulatory, antioxidant, antimicrobial, and ferroptosis-relevant agents (91, 99). These systems are potentially well suited to align axis-based interventions with the spatial and temporal complexity of periodontal lesions (91, 104).

Nanoplatforms may contribute through several complementary mechanisms. First, they may directly modulate macrophage polarization by providing immunoregulatory cues or delivering polarization-modulating agents (105). Second, they may attenuate oxidative stress and lipid peroxidation by carrying ROS-scavenging or antioxidant components, as exemplified by cerium-based systems and multifunctional composite platforms (106). Third, they may help overcome local pharmacokinetic limitations by prolonging residence time and enabling controlled release at inflamed periodontal sites (99). Gold nanoparticles, cerium oxide-based materials, and hydrogel-based composite systems may fit within this framework when used to reduce inflammatory amplification and ferroptosis-related susceptibility in a coordinated manner (91, 99, 105, 106).

Local delivery systems further support the translational rationale for this therapeutic framework. Chitosan-based hydrogels, sustained-release microparticle formulations, and targeted periodontal carriers may help maintain therapeutic concentrations within lesions while minimizing systemic exposure (95, 96, 99). Their value lies not in material novelty per se, but in disease-relevant precision, whereby immune modulation, redox control, and regenerative support can be coordinated within the same local microenvironment (96, 99, 107). Future platforms may be further refined through incorporation of stimuli-responsive release mechanisms triggered by ROS, pH, inflammatory mediators, or fluctuations in iron availability. Therefore, local delivery platforms may be important for translating axis-based interventions from mechanistic concepts to clinically translatable adjunctive periodontal strategies.

### 4.4 Exosome-based approaches as emerging strategies

Exosome-based approaches are emerging as potential strategies for immune reprogramming through cargo-dependent intercellular communication. Exosomes derived from mesenchymal stem cells and other periodontium-relevant sources can reshape macrophage polarization and regulate inflammatory signaling in recipient cells (75, 108). This approach is potentially relevant in periodontitis, where effective host modulation may require coordinated regulation of immune states and structural cell resilience (76, 108). Emerging evidence from other disease models also suggests that exosome cargo may influence ferroptosis-related pathways, although this effect remains to be directly validated in periodontal lesions (67).

In the periodontal setting, exosomes may act at multiple levels. Exosomes derived from gingival mesenchymal stem cells, periodontal ligament-derived cells, or dental pulp stem cells can suppress pro-inflammatory macrophage activity and promote pro-resolving phenotypes. They may also support a microenvironment conducive to tissue regeneration (76–78). Depending on their cargo composition, exosomes may influence pathways involving SLC7A11, GPX4, inflammatory transcriptional programs, or ROS buffering, thereby potentially modulating ferroptosis-related susceptibility in recipient cells (79). Incorporation into hydrogels or other local delivery platforms may further enhance retention and functional stability (109).

Nonetheless, enthusiasm for exosome-based approaches should be tempered by recognition of current translational barriers. Source heterogeneity, incomplete cargo standardization, variable targeting efficiency, uncertainty regarding stability in the oral environment, and limited periodontal validation of ferroptosis-related effects remain substantial challenges. Exosome-based approaches should therefore be regarded as promising but still largely preclinical tools for localized immune and redox-related modulation of the macrophage polarization–ferroptosis axis (110, 111). Their translational potential may be greatest when integrated with local biomaterial platforms that enhance retention, protection, and tissue targeting (111, 112).

### 4.5 Photobiomodulation as an adjunctive redox-modulatory approach and combination therapy

Photobiomodulation may serve as an adjunctive host-modulatory strategy by modulating mitochondrial function, ROS dynamics, and antioxidant responses within periodontal tissues (113, 114). Its therapeutic value may lie primarily in supportive integration into multimodal intervention frameworks that combine redox regulation, immune reprogramming, and local regenerative support, rather than in stand-alone use (115). This approach is potentially relevant because the macrophage polarization–ferroptosis axis is unlikely to be adequately controlled through single-node interventions alone.

In principle, photobiomodulation may help attenuate oxidative overload, support mitochondrial resilience, and reinforce endogenous antioxidant defenses (113, 114). These effects are particularly relevant in periodontitis, where redox imbalance is embedded within a broader network of inflammatory and metabolic dysfunction. When appropriately integrated with local delivery systems, antioxidant therapies, or macrophage-targeted interventions, photobiomodulation may help modulate the lesional microenvironment and support a shift from destructive persistence toward reparative remodeling (115).

Because inflammatory cytokine signaling, iron dysregulation, oxidative stress, ferroptotic injury, and impaired tissue regeneration are tightly intertwined, multimodal strategies modulating multiple nodes within the macrophage polarization–ferroptosis axis may be more biologically plausible and clinically realistic to explore than any single intervention alone (116). However, photobiomodulation should currently be interpreted as an adjunctive redox- and inflammation-modulatory approach, rather than as an established ferroptosis-specific therapy in periodontitis. Direct evidence linking photobiomodulation to ferroptosis-specific target engagement in periodontal lesions remains limited and should be addressed in future periodontal models.

## 5 Translational bottlenecks and future directions

### 5.1 Evidence hierarchy and human validation

Although the macrophage polarization–ferroptosis axis provides a useful framework for integrating immune dysregulation, iron–redox imbalance, lipid peroxidation, and periodontal tissue destruction, its translational relevance depends on stronger human validation. Current evidence remains uneven across mechanistic nodes, with relatively stronger support for oxidative stress, inflammatory cytokine enrichment, and ferroptosis-associated molecular changes, but weaker direct evidence for spatially organized macrophage–ferroptosis crosstalk in patient lesions (11, 102, 117).

Future human studies should move beyond measuring isolated markers and instead determine whether macrophage states, iron-retention programs, lipid peroxidation signals, antioxidant-defense failure, and ferroptosis-vulnerable cell populations are spatially and temporally linked within periodontal lesions. In practical terms, this requires answering three related questions: where ferroptosis-associated changes occur, which macrophage subsets are located near these regions, and whether these spatial relationships differ across destructive, resolving, and regenerative lesion states (11, 102, 118).

A stronger validation framework should therefore integrate multiplex immunophenotyping, single-cell transcriptomics, spatial transcriptomics, and redox- or iron-related molecular profiling with clinical periodontal parameters. Such approaches could clarify whether the macrophage polarization–ferroptosis axis represents a general feature of periodontitis or a lesion-specific mechanism enriched in particular patient subgroups, disease stages, or inflammatory microenvironments (118–121).

For translational studies, validation should also include mechanism-matched target-engagement biomarkers rather than relying only on downstream clinical improvement. Candidate readouts should be aligned with the proposed intervention node, including macrophage-state markers, cytokine profiles, iron-handling signals such as the hepcidin–ferroportin axis, ferroptosis-relevant redox and lipid peroxidation markers such as SLC7A11, GPX4, ACSL4, labile iron, and lipid peroxide accumulation, as well as inflammatory-resolution and tissue-repair indicators. Pairing these molecular readouts with clinical parameters such as probing depth, clinical attachment level, bleeding on probing, alveolar bone preservation, and regenerative outcomes will be necessary to determine whether a given intervention truly engages the intended macrophage–ferroptosis-related mechanism in periodontal lesions.

For specimen-specific implementation, gingival biopsies are suited to spatial and cell-resolved assessment of macrophage-state markers, SLC7A11, GPX4, ACSL4, tissue iron, and lipid peroxide accumulation, whereas gingival crevicular fluid may enable serial measurement of cytokines, iron-handling-related signals, and soluble redox or lipid-peroxidation products. The feasibility of detecting ferroptosis-associated changes in gingival crevicular fluid and the value of cell-resolved gingival profiling are supported by emerging human studies, but these proposed matrix-specific panels still require analytical and clinical validation before they can be interpreted as evidence of target engagement (11, 102, 118–121).

### 5.2 Beyond the M1/M2 dichotomy

A second major translational bottleneck is the continued reliance on the simplified M1/M2 framework. Although this dichotomy remains useful for conceptual organization, macrophage states *in vivo* are unlikely to conform neatly to two discrete categories (122, 123). In periodontal tissues, macrophage polarization is shaped by microbial stimulation, cytokine gradients, metabolic stress, and interactions with resident stromal and immune cells (18, 123). Collectively, these influences are more likely to generate a continuum of functional states rather than fixed polarization endpoints.

This issue has important therapeutic implications. Because macrophage responses are more heterogeneous than the M1/M2 model suggests, broad reprogramming strategies may produce variable effects across different disease contexts (124, 125). Some macrophage subsets may be predominantly pro-inflammatory, whereas others may be specialized for tissue clearance, angiogenesis, osteoimmune regulation, or reparative signaling (18, 124). Similarly, the consequences of ferroptosis-associated stress may differ across these subpopulations. Future research should therefore move beyond binary classification and identify which macrophage states are most closely linked to iron remodeling, oxidative injury, defective efferocytosis, and therapeutic responsiveness.

High-resolution profiling approaches are particularly needed to determine whether distinct macrophage subsets colocalize with ferroptosis-prone structural cells, osteoclast-related compartments, or regenerative niches within periodontal lesions (92, 123). Such information will be essential for refining the macrophage polarization–ferroptosis axis from a conceptual framework into a more selective and testable translational framework.

### 5.3 Cell-type specificity and therapeutic selectivity

The therapeutic significance of ferroptosis modulation in periodontitis is unlikely to be uniform across all target cell populations. In structural cell types, particularly periodontal ligament-derived cells and gingival fibroblasts, ferroptotic injury is likely to compromise tissue homeostasis, matrix integrity, and regenerative capacity. In these contexts, suppression of ferroptosis may help preserve tissue function and support repair (25, 89). However, the situation is likely more complex in osteoclast-related pathways or highly activated inflammatory immune populations, where context-dependent modulation may influence bone resorption and lesion dynamics in distinct ways (126).

Comparable issues of therapeutic selectivity also apply to macrophage targeting. Strategies designed to limit pro-inflammatory macrophage activity may be beneficial in chronic destructive lesions; however, excessive or poorly timed immune suppression could impair pathogen control or tissue clearance (126, 127). The future of host-modulatory therapy in periodontitis therefore lies not in indiscriminate inhibition of inflammation or ferroptosis, but in selective interventions tailored to cellular identity, lesion stage, and therapeutic objectives (128).

This perspective also cautions against viewing ferroptosis as uniformly detrimental. Its biological impact may depend on the specific cell populations affected—whether resident reparative cells or osteoclast-related effectors—and may also vary with the timing of intervention during disease progression (22, 90). Accordingly, precise therapeutic strategies will need to account for both cell type and disease stage.

### 5.4 Toward precision host-modulatory therapy

Future progress in this field will depend on the development of precision host-modulatory approaches capable of integrating immune reprogramming, redox regulation, and local regenerative support. Because periodontitis is sustained by a network of interacting processes rather than a single dominant pathway, strategies developed within the macrophage polarization–ferroptosis framework will likely need to incorporate multiple functions, including cytokine modulation, restoration of antioxidant defenses, regulation of iron handling, and selective support of reparative cell populations (95, 129).

Local delivery platforms may be particularly useful in this context. Stimuli-responsive systems that respond to ROS burden, inflammatory mediators, pH changes, or fluctuations in iron availability may enable stage-specific and lesion-tailored interventions. Such platforms can improve retention within periodontal pockets, reduce systemic exposure, and facilitate coordinated release of immunomodulatory and ferroptosis-relevant agents (130, 131). Exosome-enabled delivery, biomaterial-assisted targeting, and combination strategies incorporating photobiomodulation may further expand these therapeutic possibilities as emerging adjunctive approaches (132).

Ultimately, meaningful translation will require not only deeper mechanistic insight but also standardized preclinical evaluation, reproducible delivery performance, and well-designed human studies. The next phase of research should therefore integrate mechanistic discovery with therapeutic engineering, enabling rigorous evaluation of the macrophage polarization–ferroptosis axis as a clinically translatable framework for precision host-modulatory intervention in periodontitis.

## 6 Conclusions

Current evidence supports considering macrophage polarization and ferroptosis as an emerging and biologically plausible immunometabolic framework in periodontitis. Through proposed interactions involving inflammatory cytokine networks, iron remodeling, oxidative stress, lipid peroxidation, danger signaling, defective efferocytosis, and intercellular communication, this framework may help explain how periodontal lesions shift from microbial-triggered inflammation toward persistent tissue destruction and impaired repair.

From a therapeutic perspective, the macrophage polarization–ferroptosis framework highlights multiple therapeutically targetable and candidate nodes for host-modulatory intervention, including macrophage reprogramming, ferroptosis-relevant redox and lipid peroxidation modulation, iron–redox control, biomaterial-assisted local delivery, exosome-based approaches, and multimodal combination strategies. Coordinated modulation of these nodes may help remodel the periodontal microenvironment toward inflammatory resolution, tissue repair, and regeneration, but these strategies remain incompletely validated in human periodontitis.

Important translational barriers remain. Macrophage states and ferroptotic responses are highly context dependent, and current evidence is still dominated by preclinical models. Future studies should prioritize spatially resolved human validation, standardized macrophage and ferroptosis phenotyping, mechanism-matched target-engagement biomarkers, cell-selective targeting, stage-aware intervention design, locally deliverable therapeutic platforms, and well-designed clinical studies.

A deeper understanding of this framework may provide a rationale for mechanism-based, lesion-aware, and stage-adapted host-modulatory strategies for periodontitis. Beyond periodontal disease, this framework may also offer a hypothesis-generating model for understanding how macrophage-driven inflammatory microenvironments interact with ferroptosis-related tissue injury in chronic mucosal inflammatory disorders.
